# The role of sleep quality in psychotic-like experiences

**DOI:** 10.1192/j.eurpsy.2022.912

**Published:** 2022-09-01

**Authors:** P. Simor, B. Polner, N. Báthori, P. Peigneux

**Affiliations:** 1 Université Libre de Bruxelles, Neuropsychology And Functional Neuroimaging Research Unit At Center For Research In Cognition And Neurosciences And Uni - Ulb Neurosciences Institute, Bruxelles, Belgium; 2 Eötvös Loránd University, Institute Of Psychology, Budapest, Hungary; 3 Budapest University of Technology and Economics, Department Of Cognitive Science, Budapest, Hungary

**Keywords:** sleep, psychosis, mood, anxiety

## Abstract

**Introduction:**

Impaired sleep quality is among the most common complaints in psychopathological conditions including psychotic states. The clinical relevance of sleep disruption is, however, notoriously overlooked and considered as a secondary symptom that automatically ameliorates if the mental problem is adequately treated. Nevertheless, research findings indicate that sleep quality has a causal role in the occurrence and maintenance of psychotic states, and instead of being merely the “nocturnal impact” of an underlying mental disorder, shows bidirectional associations with mental health complaints.

**Objectives:**

Although the majority of studies examined the links between sleep and psychosis by cross-sectional assessments, sleep quality and psychotic-like experiences both fluctuate from night to night and day to day, respectively, even in non-clinical populations. The prospective assessment of these variables hence allows for the analyses of the temporal (and intraindividual) associations between sleep and psychosis. In our studies, we examined the temporal, bidirectional associations between sleep quality and psychotic-like states.

**Methods:**

Across three experience sampling studies with participants from the general population ( N = 73 / 166 / 60), we assessed sleep quality and daytime psychotic-like phenomena every day for at least two weeks. Using mixed-effects models, we examined if sleep quality predicted psychotic-like experiences the following day, and also if psychotic-like experiences predicted sleep quality the following night.

**Results:**

Our findings consistently highlight the dominant direction of prediction from sleep to daytime psychotic-like experiences, whereas the inverse direction is not supported by enough evidence.

**Conclusions:**

Individuals at risk for psychosis could benefit from sleep-specific interventions that could be integrated into treatment protocols.

**Disclosure:**

No significant relationships.

